# Genome-guided prebiotic fermentation generates anti-virulence metabolites against *Pseudomonas aeruginosa*

**DOI:** 10.3389/fmicb.2026.1948465

**Published:** 2026-09-15

**Authors:** Rasha Elsayim, Monerah S. M. Alqahtani, Malak Muhayya Alosaimi, Alaa Alnoor Alameen, Dalia Al Sarar, Ejlal Mohamed Abdullah, Esra’a Abudouleh

**Affiliations:** 1 Department of Botany and Microbiology, College of Science, King Saud University, Riyadh, Saudi Arabia; 2 Department of Biology, Faculty of Science, King Khalid University, Abha, Saudi Arabia; 3 Department of Oral Medicine and Diagnostic Sciences, College of Dentistry, King Saud University, Riyadh, Saudi Arabia; 4 Department of Pharmacology and Toxicology, College of Pharmacy, King Saud University, Riyadh, Saudi Arabia; 5 Department of Biochemistry, College of Science, King Saud University, Riyadh, Saudi Arabia

**Keywords:** anti-virulence, Arabic gum, baobab, *Bifidobacterium longum*, biofilm, CAZyme, cell-free supernatant, *lactiplantibacillus plantarum*

## Abstract

**Background:**

The rise of multidrug-resistant *Pseudomonas aeruginosa* has heightened interest in microbiome-based anti-virulence strategies that mitigate pathogenicity without directly affecting bacterial survival. This study examined whether the fermentation of Arabic gum and baobab by probiotic bacteria produces metabolites that reduce *P. aeruginosa* virulence.

**Methods:**

The carbohydrate-active enzyme profiles of *Bifidobacterium longum*, *Lactiplantibacillus plantarum*, and *P. aeruginosa* were compared to predict their ability to utilize plant glycans. Probiotic growth, viable counts, and acidification were measured following supplementation with Arabic gum, baobab, or glucose. The effects of untreated, neutralized, and pH-matched cell-free supernatants on *P. aeruginosa* growth, biofilm formation, adhesion, and twitching motility were evaluated.

**Results:**

*L. plantarum* and *B. longum* exhibited 54 and 53 glycoside hydrolases, respectively, compared to 29 in *P. aeruginosa*, suggesting a superior probiotic capacity for plant-glycan utilization. Arabic gum at 2% significantly enhanced *B. longum* viability by approximately 0.27 log_10_ CFU mL^−1^ (*P* = 0.0058) and resulted in the greatest pH reduction, whereas glucose increased *L. plantarum* growth by approximately 0.58 log_10_ CFU mL^−1^ (*P* = 0.0019). Supernatants derived from Arabic gum exhibited the strongest activity, nearly completely inhibiting biofilm formation, reducing adhesion by approximately 54%, and significantly suppressing twitching motility. Baobab demonstrated measurable but generally weaker prebiotic and anti-virulence effects. These findings indicate that the biological effects were contingent on the fermented substrate and the resulting metabolite profile. Neutralization reduced several inhibitory effects, while pH-matched controls largely replicated the growth suppression observed with the corresponding CFS. This indicates that fermentation-associated acidification was a primary determinant of antibacterial activity. The residual activity observed in certain neutralized CFS preparations suggests that additional non-acidic factors may contribute under specific conditions.

**Conclusion:**

Genome-informed glycan-utilization predictions identified substrates capable of producing probiotic metabolites that attenuate multiple *P. aeruginosa* virulence phenotypes. Arabic gum shows particular promise for microbiome-based control of multidrug-resistant *P. aeruginosa.*

## Graphical Abstract

**Figure:**
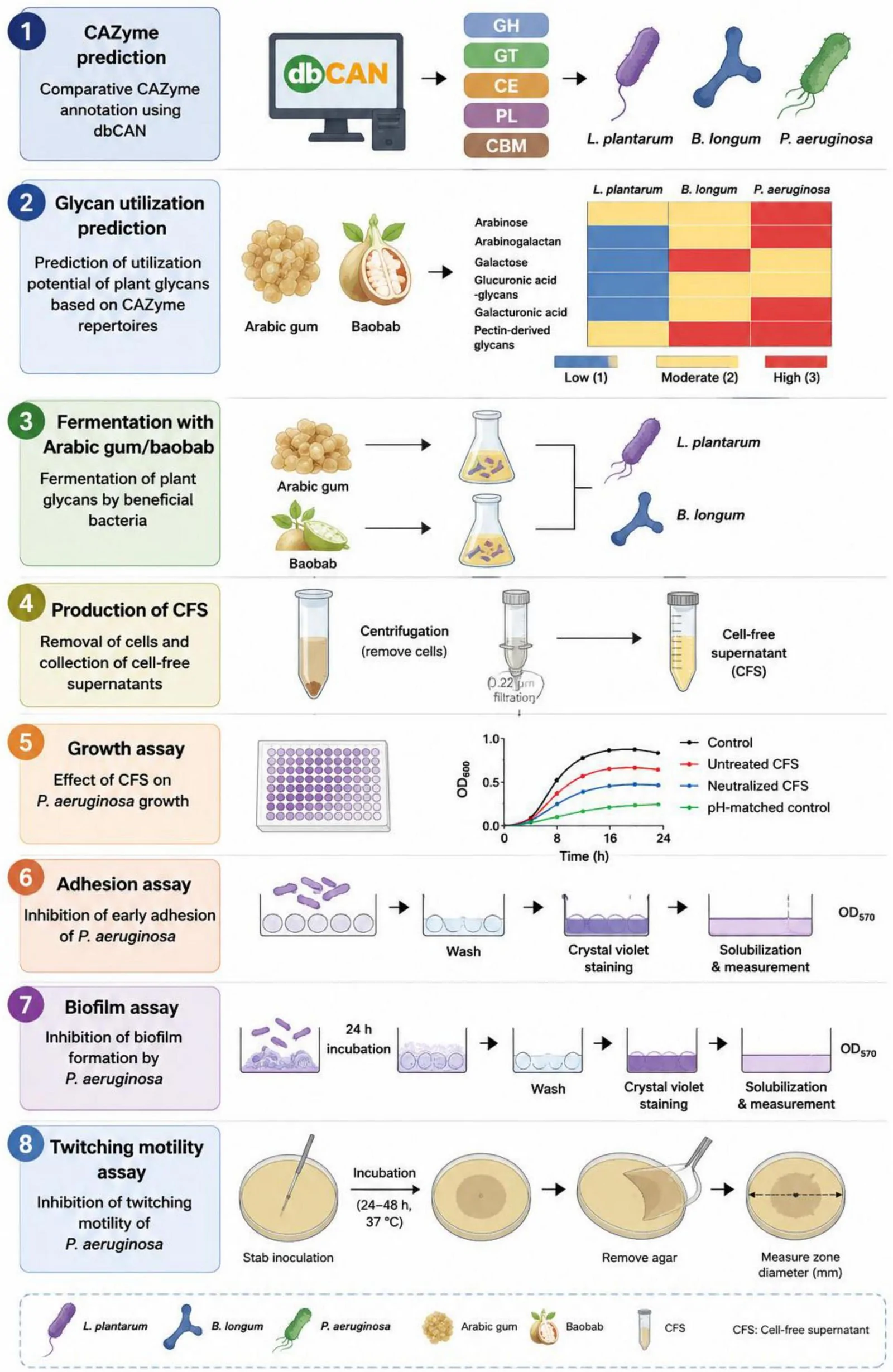
The research process demonstrates the prediction of plant glycan utilization using CAZymes, the fermentation of Arabic gum and baobab by *L. plantarum* and *B. longum*, the creation of cell-free supernatants (CFS) derived from probiotics, and the assessment of their impact on the growth, adhesion, biofilm development, and twitching motility of *P. aeruginosa*.

## 1 Introduction

*Pseudomonas aeruginosa*, a Gram-negative opportunistic pathogen, is a significant contributor to both healthcare-associated and community-acquired infections, particularly affecting immunocompromised individuals and those with pre-existing medical conditions. It is commonly linked to infections of the respiratory tract, chronic wounds, urinary tract, bloodstream, and medical devices. The treatment of these infections is often complicated by the pathogen’s exceptional adaptive capacity and inherent resistance to multiple antimicrobial agents (Moradali et al., 2017). Beyond its antimicrobial resistance, *P. aeruginosa* is clinically significant due to its numerous virulence factors that enable survival in diverse and hostile environments. Notably, biofilm formation is a critical pathogenic trait, facilitating bacterial persistence, protection from host immune responses, and increased tolerance to antimicrobial therapy (Al-Dahmoshi et al., 2021; Yin et al., 2022). As a result, infections associated with biofilm-forming *P. aeruginosa* are often chronic, recurrent, and challenging to eradicate, significantly contributing to treatment failures and increased healthcare burdens (Das, 2021). Due to its multidrug resistance, adaptability, and biofilm-forming ability, * P. aeruginosa* is recognized as a high-priority antimicrobial-resistant pathogen and remains a major global public health concern (Moradali et al., 2017; Yin et al., 2022).

Adhesion and biofilm formation are fundamental virulence mechanisms that facilitate *P. aeruginosa*’s colonization of host tissues and abiotic surfaces, thereby establishing persistent infections. The development of biofilms is a multistage process, initiating with reversible surface attachment, progressing to irreversible adhesion, microcolony formation, matrix production, maturation, and culminating in dispersal (Yang et al., 2011). The biofilm matrix consists of extracellular polymeric substances, such as polysaccharides, extracellular DNA, and proteins, which provide structural support and shield bacterial cells from environmental stressors and antimicrobial agents (Janissen et al., 2015; Mann and Wozniak, 2012). Phenotypes related to adhesion, including cell-surface attachment and twitching motility, are strongly linked to robust biofilm formation in clinical *P. aeruginosa* isolates, underscoring the significance of assessing adhesion, biofilm biomass, and motility as interconnected virulence traits (Patel and Gajjar, 2022). Consequently, strategies that disrupt early adhesion or biofilm development may serve as effective anti-virulence approaches to limit *P. aeruginosa* persistence. Bacterial adhesion and surface-associated motility are critical factors in the establishment and maturation of biofilms. Disrupting these initial colonization processes can potentially diminish bacterial persistence and reduce virulence without necessarily compromising bacterial viability (Patel and Gajjar, 2022). Therefore, adhesion, biofilm formation, and twitching motility were selected as key virulence-associated phenotypes for evaluation in the present study.

Traditional antimicrobial therapies are designed to either inhibit bacterial growth or directly eliminate bacterial cells. However, the rising incidence of antimicrobial resistance has prompted a shift toward anti-virulence strategies that target pathogenic behaviors rather than bacterial viability (Liao et al., 2022). These strategies aim to disrupt key virulence factors, such as adhesion, biofilm formation, quorum sensing, and host colonization, thereby diminishing bacterial pathogenicity while applying less selective pressure for resistance development (Fu et al., 2021). In the case of *P. aeruginosa*, early adhesion and biofilm formation are crucial for establishing persistent infections, rendering them appealing targets for therapeutic intervention. Unlike conventional antibiotics, anti-adhesion and anti-biofilm agents can impede bacterial attachment, colonization, and biofilm maturation without necessarily exerting strong bactericidal effects (Hanot et al., 2025). As a result, inhibiting adhesion-related phenotypes, including biofilm formation and twitching motility, has emerged as a promising approach to attenuate *P. aeruginosa* virulence and enhance the management of chronic biofilm-associated infections.

Arabic gum and baobab are polysaccharide-rich dietary fibers derived from plants, garnering significant interest as natural prebiotic substrates (Duysburgh et al., 2024; Foltz et al., 2021). Arabic gum primarily consists of highly branched arabinogalactan polysaccharides, while *baobab* fruit pulp is rich in pectic polysaccharides, including structures abundant in galacturonic acid and other fermentable carbohydrates (Duysburgh et al., 2024; Fu et al., 2021). Due to their intricate glycan composition, both materials can act as fermentable substrates for beneficial gut microorganisms, thereby influencing microbial metabolic activity. Recent in vitro studies using human gut models have demonstrated that Arabic gum and baobab fiber can modulate gut microbial composition and fermentation profiles. These fibers promote the growth and activity of beneficial bacterial populations and alter microbial metabolite production. The findings suggest their potential application as microbiome-targeted dietary substrates that stimulate beneficial bacterial metabolism and produce bioactive fermentation products. Thus, Arabic gum and baobab are promising candidates for exploring microbiome-mediated strategies for pathogen control and virulence attenuation (Duysburgh et al., 2024; Foltz et al., 2021).

*Lactiplantibacillus plantarum* and *B. longum* were chosen as models of beneficial microbiota due to their classification within significant probiotic-associated bacterial groups. These species have been utilized in research examining adhesion, biofilm formation, and metabolite-mediated biological activity (Liu et al., 2021; Racioppo et al., 2023). Probiotic-derived cell-free supernatants (CFS) offer a cell-free approach to studying microbiome-mediated effects, as they contain soluble metabolites released during bacterial growth or fermentation. These metabolites may include organic acids, short-chain fatty acids, antimicrobial peptides, enzymes, and other bioactive compounds (Drumond et al., 2023; Kim et al., 2025). Previous studies have demonstrated that CFS from lactic acid bacteria, such as *L. plantarum*, can inhibit the growth and biofilm formation of *P. aeruginosa*, thereby supporting the application of probiotic-conditioned supernatants as anti-pathogen and anti-biofilm agents (Drumond et al., 2023). Similarly, research involving *Bifidobacterium*-derived CFS has shown that *B. longum* supernatants contain biologically active metabolites, including short-chain fatty acids, which can modulate host or inflammatory responses, underscoring the broader significance of *B. longum*-derived metabolite preparations (Kim et al., 2025; Quesada-Vázquez et al., 2025). Despite the increasing interest in microbiome-based interventions and anti-virulence strategies targeting antimicrobial-resistant pathogens, significant knowledge gaps persist. Although Arabic gum and baobab have been explored as prebiotic substrates, and metabolites derived from probiotics have shown anti-pathogenic properties, there is a paucity of studies that integrate genome-informed predictions of glycan utilization with experimental validation of microbiome-mediated anti-virulence activity against *P. aeruginosa*. Additionally, the effects of cell-free supernatants conditioned with Arabic gum and baobab on adhesion-associated phenotypes, such as biofilm formation and twitching motility, are not well characterized. Therefore, a comprehensive framework that connects CAZyme-based substrate prediction, probiotic fermentation, and microbiome-derived suppression of *P. aeruginosa* virulence is currently absent. This study sought to determine if Arabic gum and baobab can serve as plant-derived prebiotic glycans that selectively enhance beneficial bacterial metabolism while reducing virulence-associated traits in *P. aeruginosa*. To achieve this aim, the research incorporated CAZyme-based substrate prediction, probiotic fermentation, and cell-free supernatant validation to assess the impact of metabolites derived from plant glycans on bacterial growth, biofilm formation, adhesion, and twitching motility. Our hypothesis posited that Arabic gum and baobab would preferentially enhance the growth and metabolic activity of *L. plantarum* and *B. longum*. This enhancement was expected to result in the production of bioactive metabolites capable of inhibiting adhesion-associated virulence traits in *P. aeruginosa*, such as biofilm formation and twitching motility.

## 2 Methodology

### 2.1 Study design

This study utilized a combined genome-informed and experimental methodology to explore the potential of Arabic gum and baobab as prebiotic substrates. These substrates were assessed for their ability to support the metabolism of beneficial bacteria and reduce virulence-associated traits in *P. aeruginosa*. The research workflow included: (i) a comparative CAZyme-based prediction of plant glycan utilization by both beneficial bacteria and *P. aeruginosa*, (ii) the fermentation of Arabic gum and baobab by *L. plantarum* and *B. longum*, (iii) the production of probiotic-derived cell-free supernatants (CFS), and (iv) the assessment of CFS effects on *P. aeruginosa* growth, adhesion, biofilm formation, and twitching motility.

### 2.2 CAZyme-based prediction of carbohydrate utilization

The carbohydrate-utilization potential of *L. plantarum*, *B. longum*, and *P. aeruginosa* was evaluated through comparative CAZyme annotation. Genome assemblies or predicted protein sequences were sourced from the National Center for Biotechnology Information database and analyzed using the dbCAN web server. CAZyme annotation facilitated the identification of glycoside hydrolases, glycosyltransferases, carbohydrate esterases, polysaccharide lyases, and carbohydrate-binding modules. The dbCAN platform incorporates tools such as HMMER, DIAMOND, and dbCAN-sub for automated carbohydrate-active enzyme annotation. The CAZyme repertoires obtained were analyzed to forecast the potential for utilizing significant glycans derived from Arabic gum and baobab. These glycans include arabinose, arabinogalactan, galactose, glycans containing glucuronic acid, galacturonic acid, and polysaccharides derived from pectin (Yin et al., 2012; Zhang et al., 2018). The predicted potential for glycan utilization was comparatively categorized into three qualitative levels: low (+), moderate (++), and high (+++). This classification was based on the presence and relative abundance of CAZyme families associated with the degradation of each glycan across the three bacterial genomes.

### 2.3 Preparation of Arabic gum and baobab substrates

Unbranded food-grade Arabic gum and baobab fruit pulp powder were purchased from a traditional Sudanese food market located in Riyadh, Saudi Arabia. There was no information available regarding any commercial brand or manufacturer for these products. Arabic gum and baobab were tested at concentrations of 1 and 2% (w/v). Glucose at 1% (w/v) served as a positive fermentation control, while a substrate-free medium was used as the negative control. Aqueous preparations were chosen to simulate typical dietary intake of Arabic gum and baobab in the form of water-based beverages or preparations. This selection aimed to establish a solvent-free system for fermentation experiments, thereby eliminating potential confounding effects of organic extraction solvents on bacterial growth and fermentation. All substrate solutions were sterilized using 0.22-μm membrane filtration prior to use. The concentrations were chosen based on a two-level exploratory design, which allowed for a two-fold variation in substrate availability and facilitated direct comparison of both plant-derived substrates under identical fermentation conditions. Previous *in vitro* studies have indicated the fermentability and prebiotic potential of Arabic gum and baobab at gram-per-liter concentrations, although the specific concentrations and fermentation models used differ across studies. Therefore, the 1 and 2% concentrations employed in this study were intended as experimental screening levels rather than direct replications of a specific physiological dose. Evaluating both concentrations also allowed for the assessment of whether increased substrate availability resulted in concentration-dependent changes in probiotic growth, fermentation activity, and subsequent biological effects (Duysburgh et al., 2024; Foltz et al., 2021).

### 2.4 Evaluation of prebiotic selectivity and fermentation activity

*L. plantarum* (ATCC 8014), and were employed as models for beneficial bacteria. *B. longum* (LMG 11047) was cultures in *Bifidobacterium* medium (BSM) while, *L. plantarum* was cultured in MRS broth, both gut microbioms was supplemented with either 1 or 2% Arabic gum, 1 or 2% *baobab*, 1% glucose, or a substrate-free control medium. Post-incubation, bacterial growth was evaluated through OD600 measurements and viable plate counting. Serial 10-fold dilutions were prepared in sterile PBS, plated on MRS agar, and expressed as CFU/mL, subsequently converted to log_10_ CFU/mL for analysis. Fermentation activity was determined by measuring the culture pH before and after incubation using a calibrated pH meter. A decrease in pH was considered indicative of carbohydrate fermentation and the production of acidic metabolites. Substrate utilization was interpreted in accordance with the prebiotic concept, which posits that a prebiotic is selectively utilized by host microorganisms and confers a health-related benefit (Drumond et al., 2023; Foltz et al., 2021). To evaluate prebiotic selectivity, the growth responses of *L. plantarum* and *B. longum* were compared to those of *P. aeruginosa* (ATCC 27853) under identical substrate conditions. Substrates that preferentially facilitated the growth of beneficial bacteria while offering limited support for *P. aeruginosa* were deemed to possess favorable selective utilization characteristics.

### 2.5 Production of probiotic cell-free supernatants

Following fermentation, probiotic cultures were centrifuged to remove bacterial cells, and the supernatants were filtered through sterile 0.22-μm membrane filters to obtain cell-free supernatants (CFS). Three preparations were generated for each condition: untreated CFS, neutralized CFS adjusted to pH 7.0 using sterile NaOH, and pH-matched controls prepared by adjusting fresh sterile medium to the same pH as the corresponding CFS. These preparations were included to differentiate between acidity-mediated effects and those due to non-acidic probiotic metabolites. Probiotic CFS has been previously utilized to evaluate antibacterial and antibiofilm activity against *P. aeruginosa* (Duysburgh et al., 2024; Foltz et al., 2021; Gibson et al., 2017).

### 2.6 Effect of probiotic-derived cell-free supernatants on *P. aeruginosa* growth

The impact of probiotic-derived CFS on *P. aeruginosa* growth was assessed using 96-well microplates. *P. aeruginosa* suspension was combined with untreated CFS, neutralized CFS, pH-matched control, or corresponding medium/substrate controls and incubated under standard growth conditions. Bacterial growth was quantified by measuring OD600 post-incubation. Media blanks and substrate blanks were included for background correction. Percentage growth inhibition was calculated relative to the untreated *P. aeruginosa* control (Drumond et al., 2023).

### 2.7 Adhesion inhibition assay

The early adhesion of *P. aeruginosa* was evaluated using a short-incubation crystal violet-based adhesion assay. Briefly, *P. aeruginosa* was exposed to the different CFS preparations in 96-well plates and incubated for 45 min at 37°C to facilitate initial attachment. Non-adherent cells were removed by gentle washing with sterile PBS. Adherent cells were stained with crystal violet, washed to remove excess stain, and destained using acetic acid. Absorbance was measured at 600 nm. Adhesion inhibition was calculated after blank correction relative to the untreated *P. aeruginosa* control. Short-incubation adhesion assays and crystal violet staining are commonly employed to quantify early bacterial attachment (O’Toole, 2011). Ceftolozane/tazobactam (MAST ADATAB^®^, MAST Diagnostics, Mast Group Ltd., Merseyside, United Kingdom) was used as the positive antibiotic control. One tablet containing 0.4 mg ceftolozane and 0.4 mg tazobactam was dissolved in 100 mL of culture medium according to the manufacturer’s instructions, yielding final concentrations of 4 μg/mL ceftolozane and 4 μg/mL tazobactam.

### 2.8 Biofilm inhibition assay

The impact of CFS preparations on the formation of *P. aeruginosa* biofilms was evaluated using the crystal violet microtiter plate assay. *P. aeruginosa* was incubated with each treatment for 24 h in 96-well plates. Post-incubation, planktonic cells were removed, and the wells were washed with sterile PBS. The attached biofilms were then stained with 0.1% crystal violet. The bound stain was solubilized with 3% acetic acid, and absorbance was measured at 600 nm. Biofilm inhibition was determined relative to the untreated *P. aeruginosa* control, following the subtraction of appropriate blanks. The crystal violet microtiter plate method is extensively utilized for quantifying biofilm biomass (O’Toole, 2011; Stepanović et al., 2007).

### 2.9 Twitching motility assay

Twitching motility was assessed as an adhesion-associated virulence phenotype of *P. aeruginosa*. Both treated and untreated bacterial suspensions were inoculated onto twitching motility agar by stabbing through the agar to the agar–plate interface. Twitching motility was chosen due to its role in type IV pili-mediated surface movement, which is integral to early colonization, biofilm formation, and virulence in *P. aeruginosa*. Plates were incubated at 37°C, after which the agar was carefully removed to visualize and measure the interstitial twitching zone. The twitching diameter was recorded in millimeters, and percentage inhibition was calculated relative to the untreated *P. aeruginosa* control. Twitching motility is typically assessed by measuring interstitial colony expansion at the interface between the solidified medium and an abiotic surface (Ha et al., 2014; Turnbull and Whitchurch, 2014).

### 2.10 Statistical analysis

All experiments were performed in independent biological replicates (*n* = 3 per group). Continuous outcomes (OD600, biofilm inhibition %, CFU, net growth %) were analyzed using ordinary one-way ANOVA to compare multiple treatment groups, followed by Tukey’s multiple comparisons test to adjust for family-wise error. Data are presented as mean ± standard deviation (SD), and *P* < 0.05 were considered statistically significant. Significance levels are indicated in figures as *P* < 0.05, *P* < 0.01, *P* < 0.001, ^****^P < 0.0001. All analyses were performed using GraphPad Prism (version 10.6; GraphPad Software, San Diego, CA, United States).

## 3 Results

### 3.1 Genome-informed prediction of plant glycan utilization by beneficial bacteria and *P. aeruginosa*

A comparative analysis of carbohydrate-active enzymes (CAZymes) was conducted using genome-based annotations to explore the potential utilization of plant-derived glycans by beneficial bacteria and *P. aeruginosa* (Figure 1). The analysis revealed distinct differences in the distribution of CAZyme families among the microorganisms studied, indicating species-specific capacities for carbohydrate processing and metabolism. *L. plantarum* and *B. longum*, the beneficial bacteria examined, exhibited the highest abundance of glycoside hydrolases (GHs), encoding 54 and 53 GHs, respectively. In contrast, *P. aeruginosa* encoded only 29 GHs. Given the central role of GHs in degrading complex polysaccharides and oligosaccharides, the expanded GH repertoires in the probiotic strains suggest a greater genomic capacity to access and metabolize structurally diverse plant glycans. Glycosyltransferases (GTs) were the second most abundant CAZyme category across all organisms examined. *L. plantarum* possessed the highest GT abundance (Ali et al., 2009), followed closely by *P. aeruginosa* (Pokusaeva et al., 2011), while *B. longum* encoded 22 GTs. The elevated GT content in *L. plantarum* may reflect its ability to synthesize and remodel carbohydrate-containing cellular structures while adapting to diverse carbohydrate substrates. Differences were also noted in accessory CAZyme families. *P. aeruginosa* contained the highest numbers of carbohydrate esterases (CEs; *n* = 8) and was the only organism predicted to encode polysaccharide lyases (PLs; *n* = 3). In contrast, no PLs were detected in either *L. plantarum* or *B. longum*. Carbohydrate-binding modules (CBMs) were present in all three species, with *P. aeruginosa* and *B. longum* each containing four CBMs, and *L. plantarum* containing three.

**FIGURE 1:**
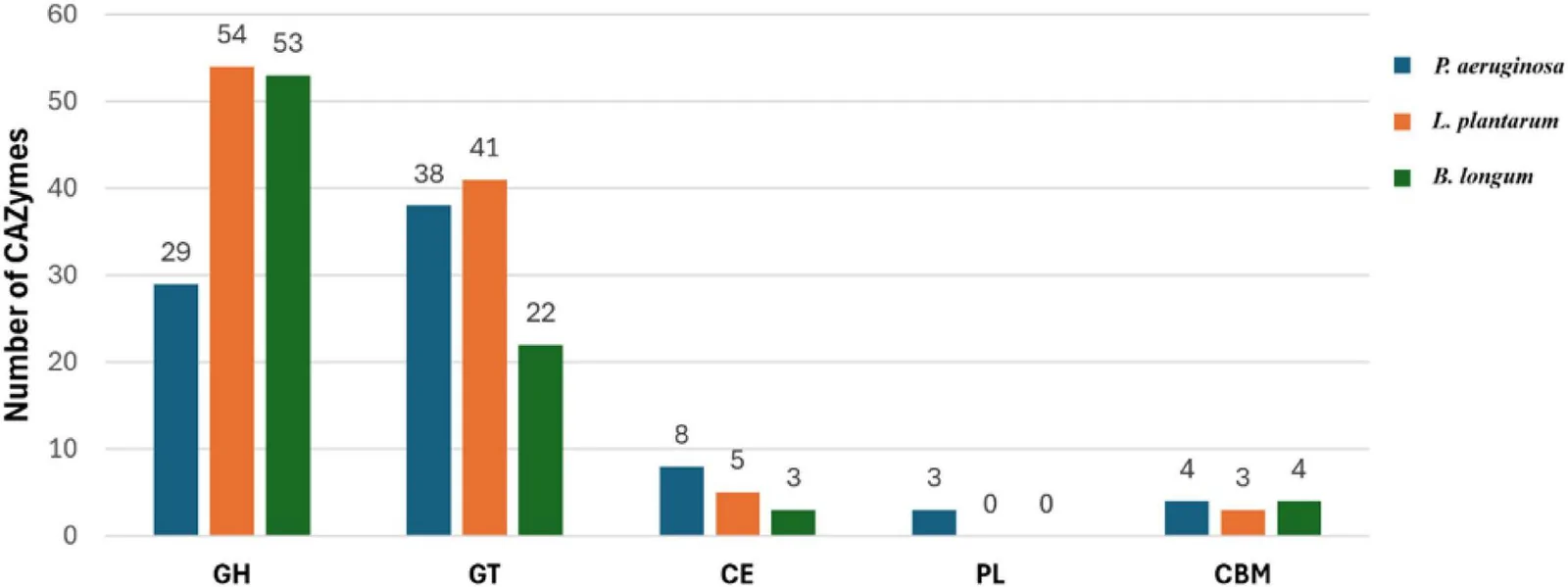
Comparison of carbohydrate-active enzyme (CAZyme) repertoires identified in the genomes of *Pseudomonas aeruginosa*, *Lactiplantibacillus plantarum*, and *Bifidobacterium longum*. Bars represent the number of genes assigned to the major CAZyme classes: glycoside hydrolases (GH), glycosyltransferases (GT), carbohydrate esterases (CE), polysaccharide lyases (PL), and carbohydrate-binding modules (CBM), as identified using the dbCAN annotation pipeline. The probiotic strains (*L. plantarum* and *B. longum*) exhibited a greater abundance of GH enzymes than *P. aeruginosa*, indicating an enhanced capacity for carbohydrate degradation and utilization, whereas *P. aeruginosa* contained a relatively higher number of PL enzymes.

In total, the genomic profiles indicate that the beneficial bacteria possess a broader repertoire of glycan-degrading enzymes, particularly glycoside hydrolases, compared to *P. aeruginosa*. These findings support the hypothesis that Arabic gum and baobab glycans may be preferentially utilized by beneficial microbiota, providing a mechanistic basis for subsequent fermentation experiments and the evaluation of microbiome-mediated effects on *P. aeruginosa* virulence-associated phenotypes.

### 3.2 Genome-informed prediction of plant glycan utilization

The comparative analysis of CAZyme repertoires identified distinct predicted utilization patterns for the primary glycan components found in Arabic gum and baobab among *B. longum*, *L. plantarum*, and *P. aeruginosa* (Table 1). *B. longum* demonstrated a high predicted utilization potential for glycans containing arabinose, arabinogalactan, galactose, and glucuronic acid. Conversely, *L. plantarum* exhibited a high predicted utilization of glycans derived from galactose, galacturonic acid, and pectin. In contrast, *P. aeruginosa* showed limited predicted utilization of arabinose and arabinogalactan, moderate utilization of glycans containing galactose, glucuronic acid, and galacturonic acid, and a high predicted utilization of pectin-derived glycans.

**TABLE 1:** Genome-informed prediction of utilization of Arabic gum and baobab *glycan* components by beneficial bacteria and *P. aeruginosa* based on CAZyme repertoire analysis.

Plant glycan component	Major source	Representative CAZyme families identified	*B. longum*	*L. plantarum*	*P. aeruginosa*
Arabinose	Arabic gum, baobab	GH43, GH51, GH121, GH127	+++	++	+
Arabinogalactan	Arabic gum	GH43, GH30, GH51	+++	++	+
Galactose	Arabic gum, baobab	GH2, GH36, GH42	+++	+++	++
Glucuronic acid-containing glycans	Arabic gum	GH30, CE families	+++	+	++
Galacturonic acid	Baobab pectin	GH28, CE1, CE2	++	+++	++
Pectin-derived glycans	Baobab	GH28, PL families, CE families	++	+++	+++

Predicted utilization potential was qualitatively categorized as low (+), moderate (++), or high (+++) based on comparative evaluation of the presence and relative representation of CAZyme families associated with each glycan across the three bacterial genomes.

### 3.3 Substrate utilization by beneficial bacteria

#### 3.3.1 Effect of substrate supplementation on the growth of *Bifidobacterium longum* and *Lactiplantibacillus plantarum*

Substrate supplementation markedly affected the growth of both probiotic strains, as evidenced by changes in optical density (OD600) post-incubation [*B. longum*: one-way ANOVA, *F*(5, 12) = 57.27, *P* < 0.0001, *R*^2^ = 0.96; *L. plantarum*: *F*(5, 12) = 13.31, *P* = 0.0002, *R*^2^ = 0.85] (Figure 2). For *B. longum*, glucose at 1% concentration resulted in the most substantial increase in culture turbidity (mean ΔOD600 = 0.239), significantly surpassing the substrate-free control, AG 1%, AG 2%, and both baobab treatments (all *P* ≤ 0.0109). Among the prebiotic substrates tested, AG 2% yielded the highest OD increase (mean ΔOD600 = 0.148), significantly greater than the untreated culture and both baobab concentrations (all *P* ≤ 0.0002). Conversely, AG 1% and BB (1 and 2%) resulted in minimal or no increase in optical density, indicating limited stimulation of bacterial biomass under these conditions. A similar pattern was observed for *L. plantarum*. Cultures without substrate showed minimal changes in OD600 (mean ΔOD600 = -0.012), whereas AG 2% (mean ΔOD600 = 0.203) and glucose 1% (mean ΔOD600 = 0.165) produced the most significant increases in bacterial growth, both significantly higher than the untreated control (*P* = 0.0004 and *P* = 0.0024, respectively). Additionally, both treatments resulted in significantly higher OD values than BB 1% and BB 2% (*P* ≤ 0.0148). Although AG 1% led to a modest increase in optical density, it did not differ significantly from the untreated culture, and neither baobab concentration significantly enhanced bacterial growth. Overall, these results suggest that glucose and AG 2% were the most effective substrates for enhancing probiotic biomass, whereas baobab supplementation had a limited effect on OD600 under the experimental conditions.

**FIGURE 2:**
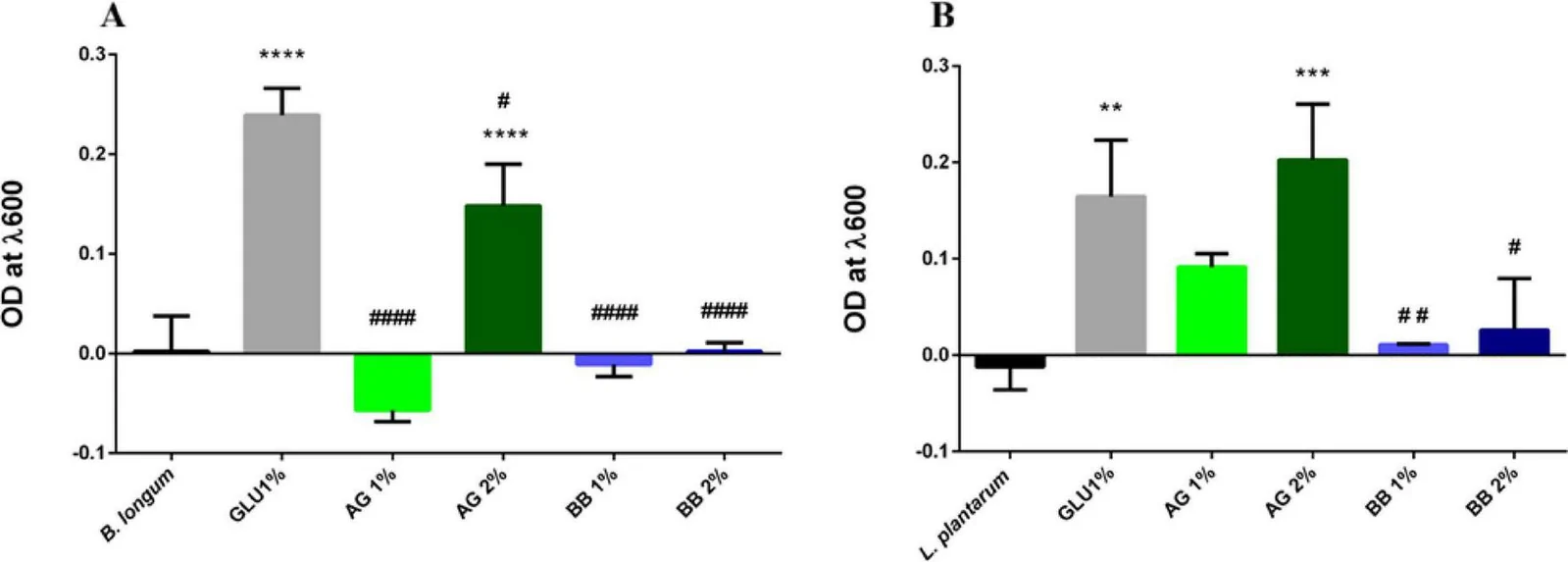
Effect of substrate supplementation on the growth of probiotic bacteria. This figure illustrates the impact of various substrates on the net growth (ΔOD600 of **(A)** *Bifidobacterium longum* and **(B)** *Lactiplantibacillus plantarum* indicate that *B. longum* was cultured in Bifidobacterium medium (BSM), whereas *L. plantarum* was cultured in MRS broth, respectively supplemented with Arabic gum (AG; 1 and 2%), baobab (BB; 1 and 2%), glucose (GLU; 1%), or a substrate-free medium (control). The net growth was determined by calculating the difference between the OD600 values measured post-incubation (Day 2) and the baseline values (Day 0). Data are presented as mean ± standard deviation (SD) derived from three independent experiments (*n* = 3). Statistical significance was evaluated using one-way ANOVA, followed by the appropriate multiple-comparison test. Asterisks denote significant differences compared to the corresponding untreated control: *P* < 0.01 (**), *P* < 0.001 (***), and *P* < 0.0001 (****). Hash symbols indicate significant differences relative to the GLU 1% group: *P* < 0.05 (#), *P* < 0.01 (##), and *P* < 0.0001 (####).

#### 3.3.2 Effect of substrate supplementation on probiotic viable counts

Substrate supplementation had a significant impact on the viable counts of *B. longum* following fermentation, as indicated by a one-way ANOVA (*F* = 8.86, *P* = 0.0097, *R*^2^ = 0.88). Among the substrates tested, 2% Arabic gum (AG) notably increased viable counts by approximately 0.27 log_10_ CFU mL^−1^ compared to the untreated control (*P* = 0.0058) (Figure 3A). Conversely, supplementation with 1% glucose, 1% AG, 1% baobab (BB), and 2% BB did not result in significant changes in viable counts relative to the control culture (all *P* > 0.41). Pairwise comparisons further revealed that 2% AG supported significantly higher *B. longum* counts than both 1% BB and 2% BB, with mean differences of approximately 0.19–0.21 log_10_ CFU mL^−1^ (*P* = 0.02 and *P* = 0.04, respectively). These findings collectively suggest that 2% Arabic gum supplementation modestly but consistently enhanced the growth of *B. longum*, whereas glucose and baobab maintained viable counts similar to those of the untreated culture. Prebiotic supplementation also significantly affected the viable counts of *L. plantarum* after fermentation, as demonstrated by a one-way ANOVA (*F* = 13.18, *P* = 0.0035, *R*^2^ = 0.92). Supplementation with 1% glucose resulted in the highest viable counts, increasing bacterial density by approximately 0.58 log_10_ CFU mL^−1^ compared to the untreated control (*P* = 0.0019) (Figure 3B). In contrast, neither Arabic gum (1 and 2%) nor baobab (1 and 2%) significantly influenced viable counts relative to the control (all *P* > 0.15). Compared to glucose, cultures supplemented with 1% AG, 2% AG, and 1% BB exhibited significantly lower viable counts, with mean differences ranging from 0.36 to 0.42 log_10_ CFU mL^−1^ (*P* ≤ 0.02). Overall, both probiotic strains remained viable in the presence of Arabic gum and baobab, although their growth responses differed according to substrate. While 2% Arabic gum preferentially enhanced the growth of *B. longum*, glucose primarily promoted the growth of L. plantarum. These modest differences in bacterial biomass suggest that subsequent variations in CFS-mediated inhibition of *P. aeruginosa* are likely attributable to differences in fermentation-derived metabolites rather than substantial changes in probiotic cell density.

**FIGURE 3:**
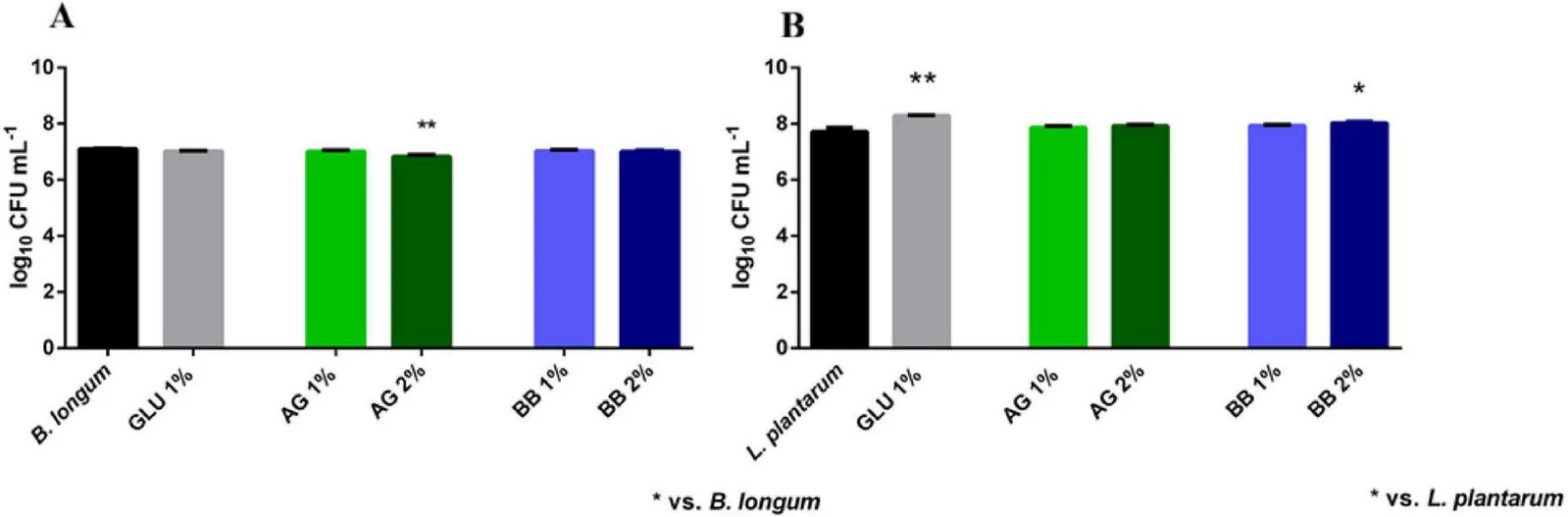
Effect of Arabic gum (AG), baobab (BB), and glucose (GLU) supplementation on the viable counts of **(A)** *Bifidobacterium longum* and **(B)** *Lactiplantibacillus plantarum* following fermentation. Viable counts were determined by serial dilution and plate counting and are expressed as log_10_ CFU mL^−1^ (mean ± SD, *n* = 3). Statistical significance was determined using one-way ANOVA followed by Tukey’s multiple-comparison test. *P* < 0.05 (*) and *P* < 0.01 (**) compared with the corresponding untreated control.

#### 3.3.3 Effect of substrate supplementation on fermentation-associated pH changes in *Bifidobacterium longum*

Prebiotic supplementation exerted a significant impact on the fermentation activity of *B. longum*, as evidenced by the final culture pH (one-way ANOVA, *F* = 44.01, *P* < 0.0001, *R*^2^ = 0.95). Figure 4A, illustrates that cultures supplemented with Arabic gum (AG) exhibited the most pronounced reduction in pH. Specifically, both 1% AG and 2% AG significantly decreased the final pH by approximately 1.5 units compared to the unsupplemented *B. longum* control (both *P* < 0.0001), indicating substantially enhanced fermentation and acid metabolite production. In contrast, baobab (BB) supplementation led to a moderate yet significant pH decrease, with both 1% BB and 2% BB reducing the final pH by approximately 0.5 units (*P* = 0.0136 and *P* = 0.0209, respectively). Supplementation with 1% glucose (GLU) resulted in only a slight pH reduction, which was not significantly different from the untreated control (*P* = 0.075). Analysis of the change in pH (ΔpH) corroborated the overall trend, confirming that Arabic gum induced the greatest acidification, followed by baobab, while glucose had minimal effect on fermentation activity. Collectively, these findings suggest that Arabic gum is the most efficiently fermented substrate by *B. longum*, leading to significantly greater acid production than either baobab or glucose. Substrate supplementation exerted a significant effect on the culture pH of *Lactiplantibacillus plantarum* post-incubation, as evidenced by a one-way ANOVA (*F* = 89.83, *P* < 0.0001, *R*^2^ = 0.97) (Figure 4B). Among the substrates evaluated, 1% glucose (GLU) resulted in the most pronounced acidification, decreasing the final pH by approximately 0.8 units relative to the untreated *L. plantarum* control (*P* < 0.0001). Conversely, supplementation with Arabic gum (AG; 1 and 2%) and 1% baobab (BB) maintained culture pH values similar to the untreated control, with no significant differences detected (*P* > 0.12). However, 2% BB led to a minor yet significant pH reduction of approximately 0.17 units compared to the control (*P* = 0.0342). Analysis of ΔpH corroborated this pattern, demonstrating that glucose induced the greatest pH reduction, while AG and BB resulted in only modest changes. Pairwise comparisons further revealed that 1% GLU was more acidifying than all other substrates, whereas 1% AG exhibited slightly greater acidification than BB treatments. Overall, these results suggest that *L. plantarum* fermentation activity was most effectively enhanced by glucose, whereas Arabic gum and baobab induced limited acidification under the conditions tested.

**FIGURE 4:**
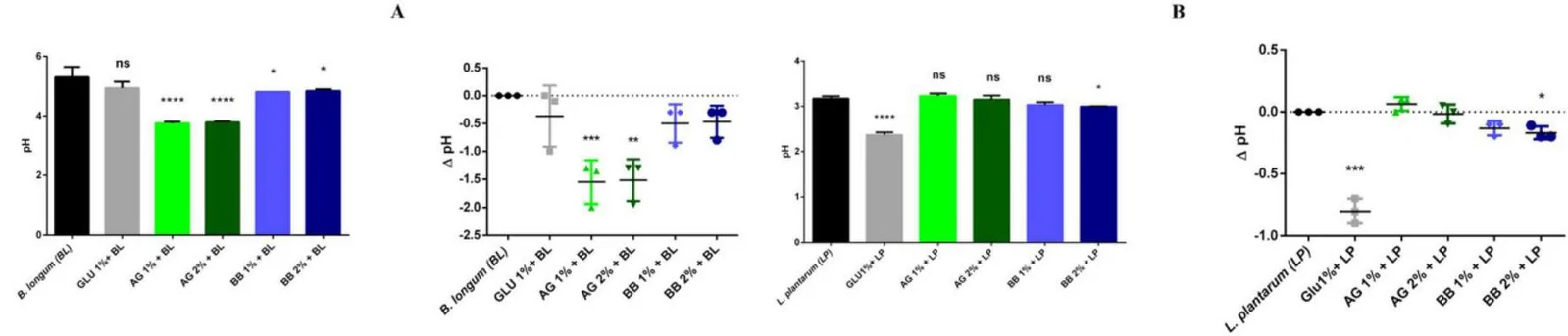
Effect of substrate supplementation on the fermentation activity of probiotic bacteria. **(A)** *B. longum* and **(B)** *L. plantarum* cultured in Bifidobacterium Selective Medium (BSM) and MRS broth, respectively, supplemented with Arabic gum (AG; 1 and 2%), baobab (BB; 1 and 2%), glucose (GLU; 1%), or without substrate (control). The left panels show the final culture pH after incubation, whereas the right panels show the change in pH (ΔpH) following fermentation. Data are presented as mean ± SD (*n* = 3). Statistical significance was determined using one-way ANOVA followed by Dunnett’s multiple-comparison test against the corresponding untreated control. Asterisks indicate significant differences relative to the corresponding untreated control: *P* < 0.05 (*), *P* < 0.01 (**), *P* < 0.001 (***), and *P* < 0.0001 (****); ns, not significant.

### 3.4 Substrate-dependent antibacterial activity of *B. longum*-derived cell-free supernatants against *P. aeruginosa*

Treatment with cell-free supernatants (CFS) derived from *B. longum* significantly influenced the net growth of *Pseudomonas aeruginosa* across various fermentation substrates and post-processing conditions, as evidenced by a one-way ANOVA (*F* = 40.78, *P* < 0.0001, *R*^2^ = 0.95) (Figure 5). The original CFS, neutralized CFS (CFS-N; adjusted to pH 7.0 with NaOH), and pH-matched controls (CFS-pH; fresh sterile medium adjusted to the same pH as the corresponding original CFS) demonstrated distinct effects on bacterial growth. Compared to the untreated *P. aeruginosa* control, the pH-matched Arabic gum-derived CFS (AG 1% CFS-pH and AG 2% CFS-pH) exhibited the most potent inhibitory activity, nearly completely suppressing bacterial growth (*P* < 0.0001). Similarly, neutralized baobab-derived CFS (BB 1% CFS-N and BB 2% CFS-N), along with BB 1% CFS-pH and BB 2% CFS-pH, significantly inhibited growth, reducing it by approximately 65–95% relative to the untreated control (*P* ≤ 0.001). Among the untreated CFS preparations, AG 2% CFS significantly inhibited *P. aeruginosa*, whereas AG 1% CFS, BB 1% CFS, BB 2% CFS, and glucose-derived CFS did not show significant differences compared to the pathogen control (*P* > 0.05). In contrast, AG 1% CFS-N, BB 2% CFS, and GLU 1% CFS-N exhibited negative inhibition values, indicating a propensity to enhance bacterial growth rather than suppress it.

**FIGURE 5:**
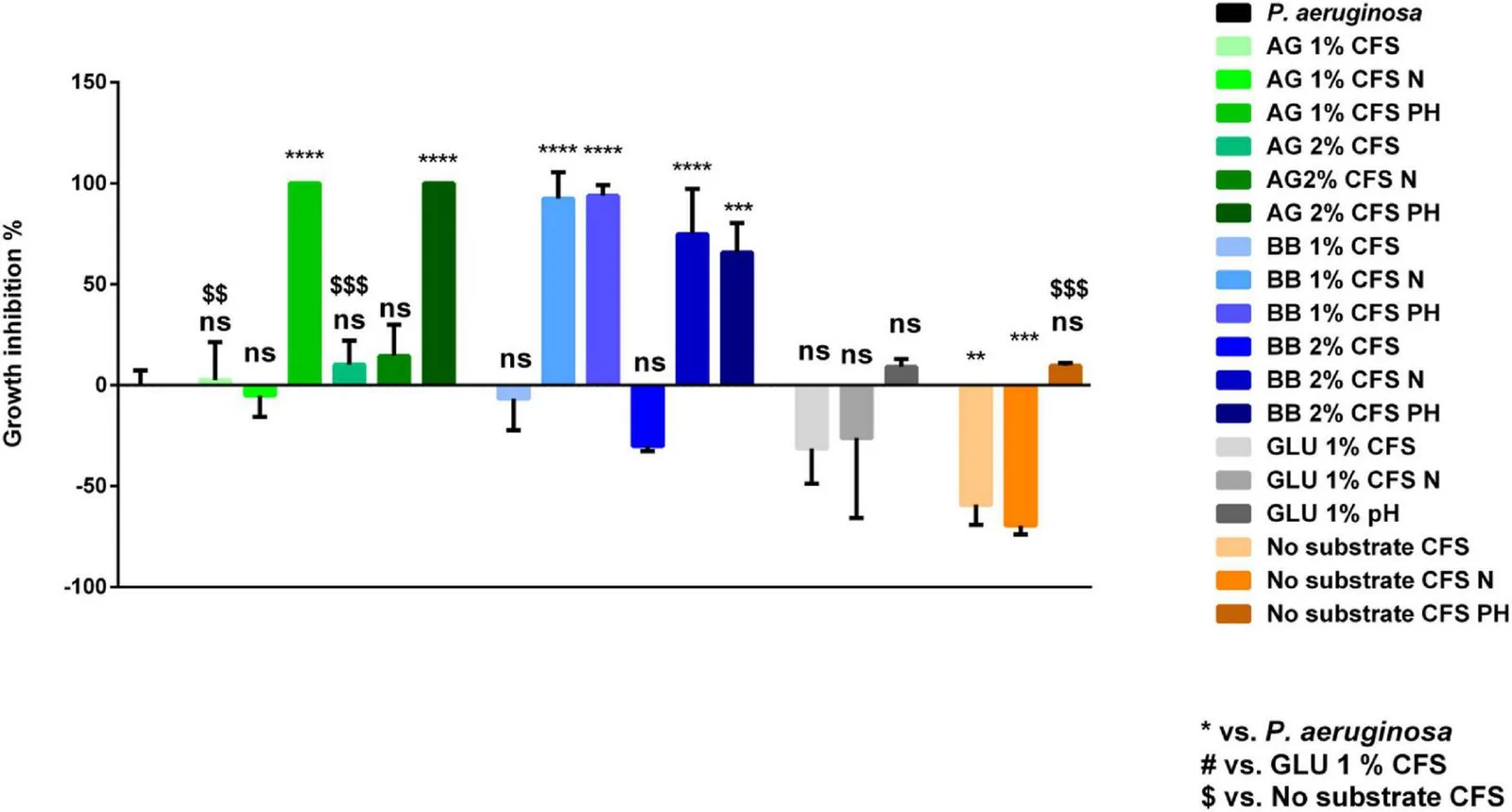
Effect of *B. longum*-derived cell-free supernatants (CFS) on the net growth of *P. aeruginosa*. CFS were obtained after fermentation with Arabic gum (AG), baobab (BB), glucose (GLU), or without substrate. CFS-N represents cell-free supernatants neutralized to pH 7.0 using 1 M NaOH, whereas CFS-pH represents fresh sterile medium adjusted to the same pH as the corresponding original CFS (pH-matched control). Data are presented as mean ± SD (*n* = 3). Data are presented as mean ± SD (*n* = 3). Statistical significance was determined using one-way ANOVA followed by Tukey’s multiple-comparison test. Asterisks indicate significant differences relative to the untreated *P. aeruginosa* control: *P* < 0.01 (**), *P* < 0.001 (***), and *P* < 0.0001 (****). Dollar symbols indicate significant differences relative to the no-substrate CFS group: *P* < 0.01 ($$) and *P* < 0.001 ($$$); ns, not significant.

Within each substrate, the effect of neutralization was substrate-dependent. Neutralization (CFS-N) nearly abolished the inhibitory activity of Arabic gum-derived CFS, whereas the corresponding pH-matched controls (CFS-pH) retained or enhanced the inhibitory effect. Within each substrate, the effect of neutralization was contingent upon the substrate. Neutralization almost completely eliminated the inhibitory activity of Arabic gum-derived CFS, whereas the corresponding pH-matched controls maintained strong inhibition. This observation suggests that the acidic environment alone was largely responsible for the growth-suppressive effect associated with these preparations. Conversely, neutralized baobab-derived CFS preserved significant inhibitory activity, indicating that additional non-acidic components may contribute to growth inhibition under these specific fermentation conditions. However, as the responsible compounds were not chemically characterized, this possibility remains tentative. The glucose-derived preparations demonstrated limited antibacterial activity overall. Neither the original glucose CFS nor the neutralized glucose CFS significantly inhibited *P. aeruginosa*, while the glucose pH-matched control produced only a modest inhibitory effect. Similarly, the no-substrate CFS and its neutralized derivative (No-substrate CFS-N) failed to inhibit bacterial growth and instead showed a tendency to promote growth, whereas the pH-matched no-substrate control (No-substrate CFS-pH) produced a small but significant inhibitory effect. Collectively, these findings indicate that the antibacterial activity of *B. longum*-derived metabolites against *P. aeruginosa* is highly substrate-dependent and is differentially influenced by neutralization and pH matching. The strongest inhibition was observed with Arabic gum-derived pH-matched CFS and baobab-derived neutralized or pH-matched CFS, whereas untreated glucose-derived CFS and no-substrate fermentations exhibited minimal or no antibacterial activity.

### 3.5 Effect of *L. plantarum*-derived cell-free supernatants on the net growth of *P. aeruginosa*

The net growth of *P. aeruginosa* was significantly affected by *L. plantarum*-derived cell-free supernatants (CFS) produced under various fermentation substrates and post-processing conditions, as indicated by a one-way ANOVA (*F* = 40.59, *P* < 0.0001, *R*^2^ = 0.95) (Figure 6). Compared to the untreated *P. aeruginosa* control, several *L. plantarum*-derived CFS preparations demonstrated marked inhibitory activity. Specifically, the original no-substrate CFS, BB 1% CFS, and BB 2% CFS achieved nearly complete inhibition of bacterial net growth, with approximately 100% inhibition (*P* < 0.0001). Similarly, the original Arabic gum-derived CFS (AG 1% and AG 2%), the pH-matched Arabic gum preparations (AG 1% CFS-pH and AG 2% CFS-pH), the pH-matched baobab preparations (BB 1% CFS-pH and BB 2% CFS-pH), and the GLU 1% CFS-pH preparation significantly reduced bacterial growth, with inhibition ranging from approximately 52–94% relative to the pathogen control (*P* ≤ 0.0031). In contrast, neutralized CFS (CFS-N) generally exhibited minimal or no inhibitory activity. Neutralized preparations derived from Arabic gum, baobab, glucose, and the no-substrate fermentation were either not significantly different from the untreated control or showed a trend toward increased bacterial growth (*P* > 0.05). These results suggest that neutralization markedly reduced the inhibitory activity observed in the original CFS preparations. Within individual substrate groups, direct comparisons revealed that neutralization consistently diminished the anti-*P. aeruginosa* activity of *L. plantarum*-derived CFS. For instance, neutralization of the AG 1% and AG 2% CFS significantly increased bacterial net growth compared to their corresponding untreated CFS preparations (*P* < 0.0001). Similar reductions in inhibitory activity were observed following neutralization of the baobab-derived CFS. Conversely, pH-matched controls generally retained inhibitory activity comparable to, or greater than, the corresponding original CFS, particularly for Arabic gum, baobab, glucose, and the no-substrate preparations. Overall, *L. plantarum*-derived CFS exhibited substantial anti-*P. aeruginosa* activity under several fermentation conditions, whereas pH neutralization largely abolished this inhibitory effect. In contrast, pH-matched controls generally maintained strong inhibitory activity, indicating that the biological activity of the fermented preparations was highly dependent on post-fermentation processing. Collectively, these findings suggest that the acidification associated with fermentation was the primary factor influencing the growth-inhibitory activity of preparations derived from *L. plantarum.*

**FIGURE 6:**
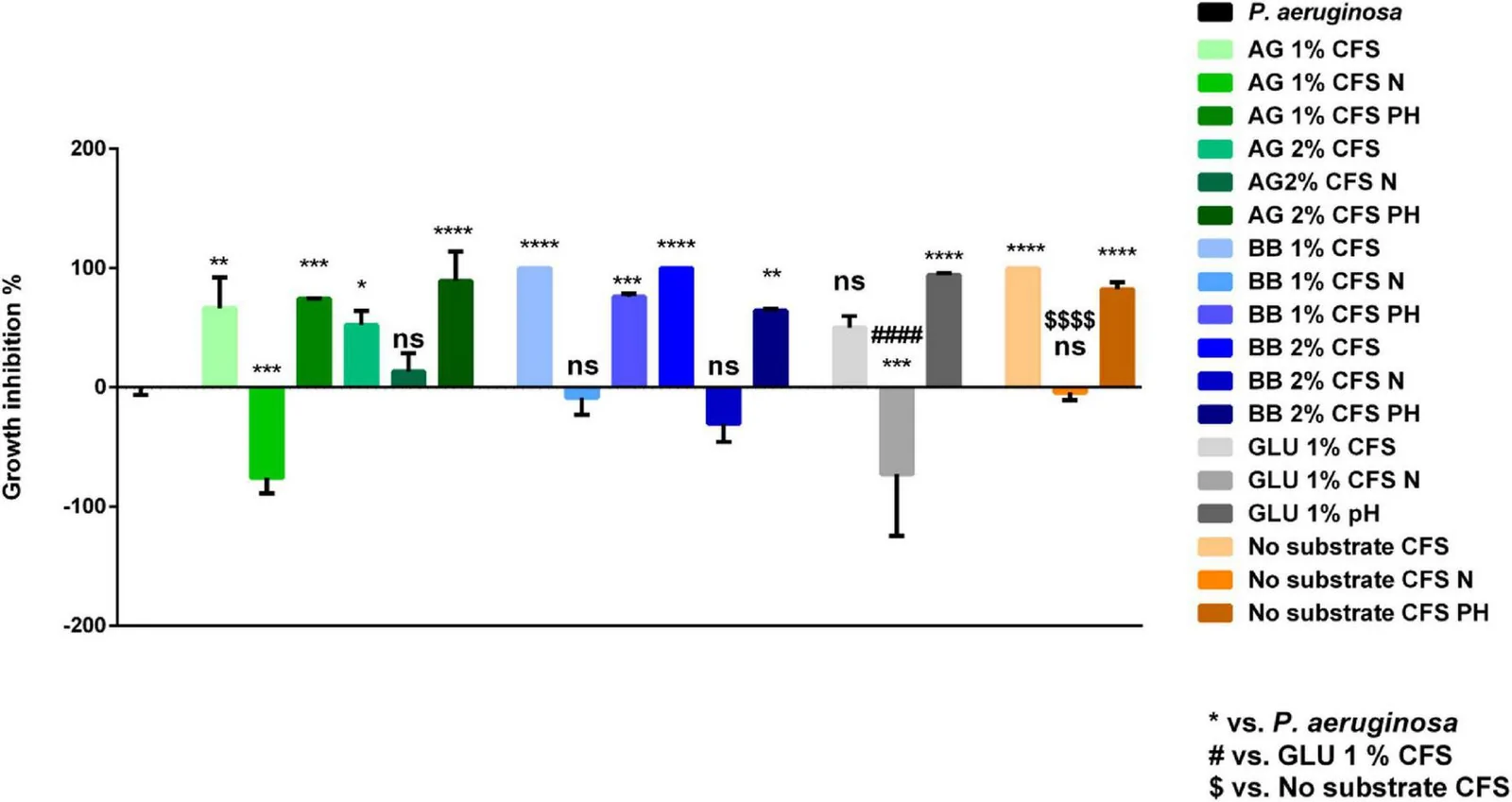
Effect of *Lactiplantibacillus plantarum*-derived cell-free supernatants (CFS) on the net growth of *Pseudomonas aeruginosa*. Data are expressed as mean ± standard deviation (SD) with a sample size of three (*n* = 3). Statistical significance was assessed using a one-way analysis of variance (ANOVA), followed by Tukey’s multiple-comparison test. Asterisks denote significant differences compared to the untreated *P. aeruginosa* control group: *P* < 0.05 (*), *P* < 0.01 (**), *P* < 0.001 (***), and *P* < 0.0001 (****). Hash symbols indicate significant differences relative to the GLU 1% CFS group: *P* < 0.0001 (####). Dollar symbols represent significant differences compared to the no-substrate CFS group: *P* < 0.0001 ($$$$); ns indicates not significant.

### 3.6 Arabic gum exhibits superior inhibition of *P. aeruginosa* biofilm formation

Prebiotic supplementation had a significant impact on *P. aeruginosa* biofilm formation, as evidenced by a one-way ANOVA (*F* = 157.1, *P* < 0.0001, *R*^2^ = 0.99) (Figure 7). The biofilm biomass, quantified through crystal violet staining (OD600), exhibited notable differences across the treatments evaluated. Arabic gum (AG) at concentrations of 1 and 2% nearly completely inhibited biofilm formation, reducing the OD600 value by approximately 0.49 units (*P* < 0.0001) compared to the untreated control. There was no significant difference between AG 1% and AG 2%, indicating that increasing the concentration from 1 to 2% did not enhance antibiofilm activity. Conversely, baobab (BB) supplementation resulted in a moderate yet significant reduction in biofilm biomass, with BB 1% and BB 2% decreasing OD600 by approximately 0.16 and 0.10 units, respectively (*P* ≤ 0.0067). Glucose supplementation yielded a similar reduction. Notably, the antibiofilm activities of both BB concentrations and glucose did not significantly differ from the antibiotic-treated group (all *P* > 0.22), suggesting comparable levels of biofilm suppression. Direct comparisons among treatments further underscored the superior efficacy of Arabic gum. Both AG concentrations resulted in significantly lower biofilm biomass than BB 1%, BB 2%, glucose, and the antibiotic control (all *P* < 0.0001), establishing Arabic gum as the most effective intervention for preventing *P. aeruginosa* biofilm development. When expressed as percentage biofilm inhibition, the data revealed a consistent pattern. AG 1% and AG 2% achieved nearly 100% inhibition of biofilm formation, whereas BB, glucose, and the antibiotic exhibited only moderate inhibition, ranging from approximately 20–33%. No significant differences were observed among BB 1%, BB 2%, glucose, and the antibiotic treatment (*P* > 0.13), indicating comparable antibiofilm efficacy among these interventions. Collectively, these findings demonstrate that Arabic gum was substantially more effective than baobab, glucose, and the antibiotic control in suppressing *P. aeruginosa* biofilm formation. The absence of additional benefit at 2% compared to 1% suggests that the maximal antibiofilm effect of Arabic gum was achieved at the lower concentration.

**FIGURE 7:**
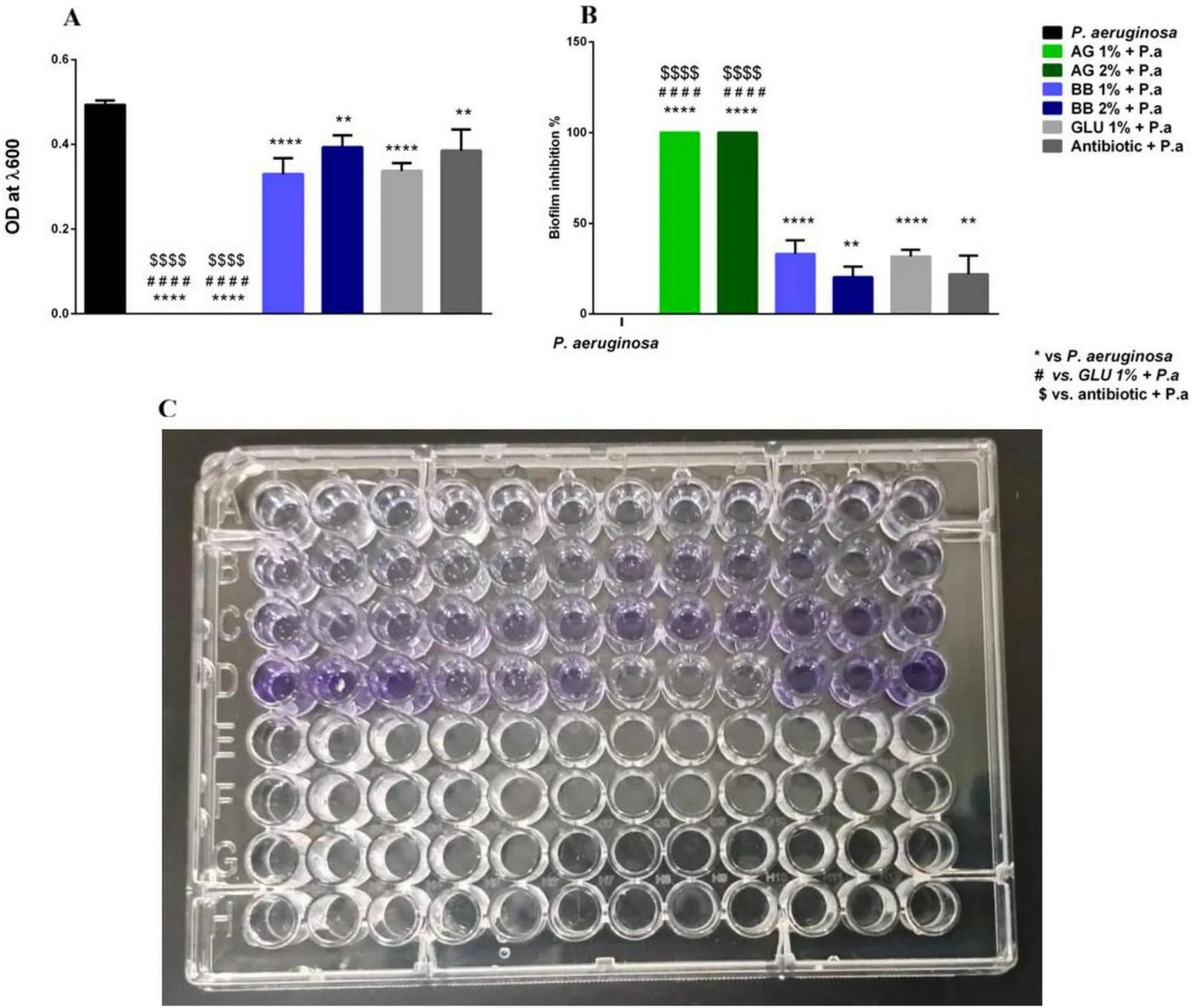
Effect of Arabic gum (AG), baobab (BB), glucose (GLU), and antibiotic treatment on *Pseudomonas aeruginosa* biofilm formation. **(A)** Biofilm biomass quantified by crystal violet staining and expressed as absorbance at OD_600_. **(B)** Percentage inhibition of biofilm formation relative to the untreated *P. aeruginosa* control. **(C)** Representative photograph of the crystal violet-stained 96-well microtiter plate following the biofilm formation assay. Data are presented as mean ± SD (*n* = 3). Statistical significance was determined using one-way ANOVA followed by Tukey’s multiple-comparison test. Asterisks indicate significant differences relative to the untreated *P. aeruginosa* control: *P* < 0.01 (**) and *P* < 0.0001 (****). Hash symbols indicate significant differences relative to the GLU 1% group: *P* < 0.0001 (####). Dollar symbols indicate significant differences relative to the antibiotic-treated group: *P* < 0.0001 ($$$$); ns, not significant.

### 3.7 Substrate-dependent modulation of *Pseudomonas aeruginosa* adhesion by prebiotic supplementation

Substrate supplementation markedly affected the adhesion of *P. aeruginosa* to abiotic surfaces, as evidenced by a one-way ANOVA (*F* = 13.50, *P* < 0.0001, *R*^2^ = 0.85) (Figure 8). Analyses of crystal violet absorbance and percentage inhibition revealed that the supplementation effect was strongly dependent on the substrate. Compared to the untreated control, Arabic gum at concentrations of 1 and 2% significantly reduced bacterial adhesion by approximately 54 and 51%, respectively (*P* < 0.05). The inhibitory effects of these two concentrations of Arabic gum were comparable and did not significantly differ from the antibiotic-treated group, suggesting that Arabic gum inhibited the initial attachment of *P. aeruginosa* with efficacy similar to that of the antibiotic control. Baobab supplementation demonstrated a weaker effect; although BB 1% and BB 2% reduced adhesion by approximately 29 and 21%, respectively, these reductions were not statistically significant compared to the untreated control (*P* > 0.05), indicating only modest inhibition of bacterial attachment. In contrast, glucose supplementation increased bacterial adhesion, resulting in higher crystal violet absorbance than the untreated control and significantly differing from both Arabic gum treatments and the antibiotic control. Pairwise comparisons further confirmed that glucose-treated cultures exhibited significantly greater adhesion than AG 1%, AG 2%, BB 1%, and BB 2% (*P* ≤ 0.0085), indicating that glucose favored surface colonization rather than preventing bacterial attachment. Overall, these findings suggest that Arabic gum was the most effective substrate for preventing the initial adhesion of *P. aeruginosa*, exhibiting inhibitory activity comparable to the antibiotic control. Conversely, baobab had limited effects, while glucose promoted bacterial attachment, underscoring the substrate-dependent nature of the adhesion response.

**FIGURE 8:**
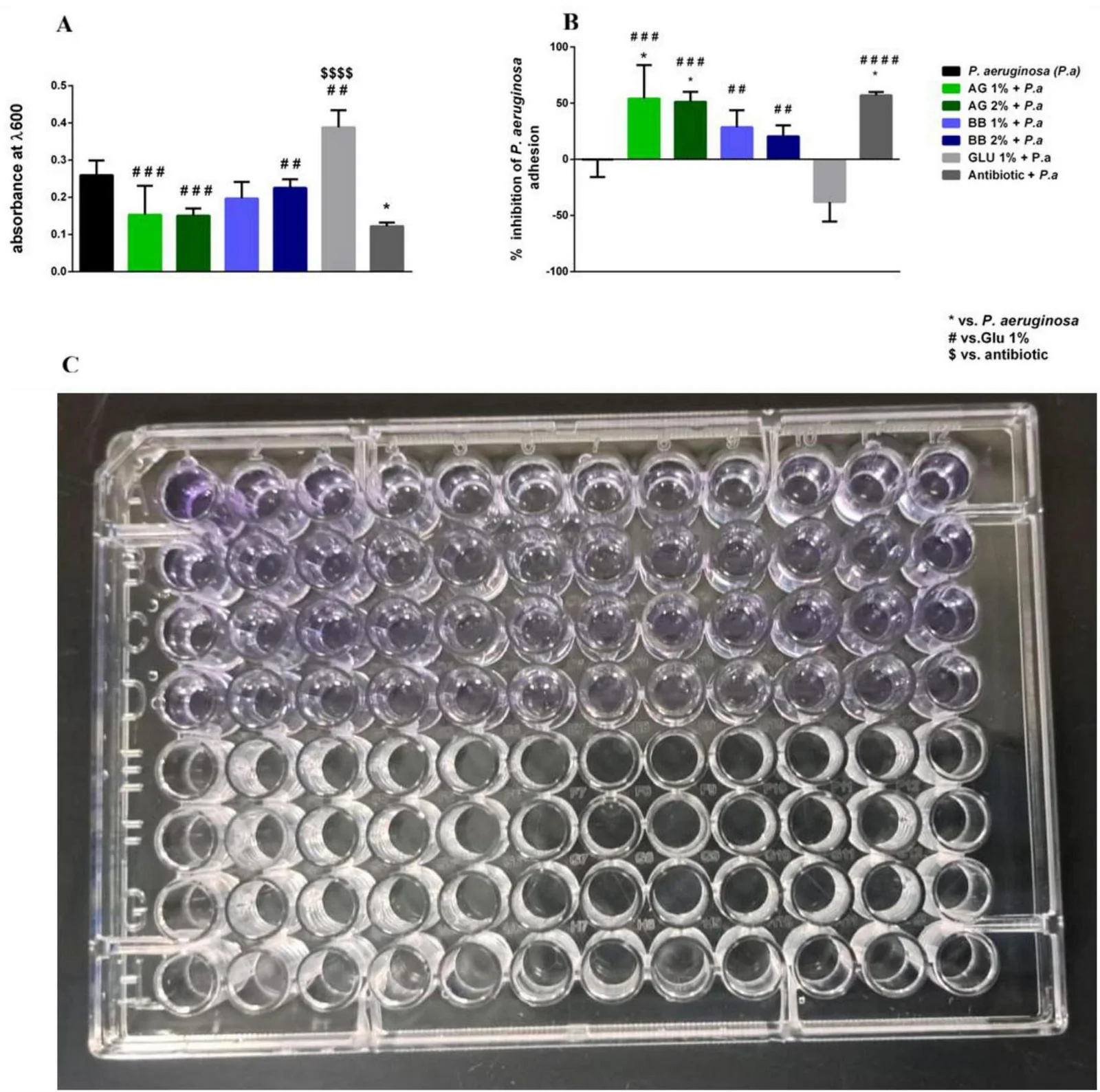
Effect of Arabic gum (AG), baobab (BB), glucose (GLU), and antibiotic treatment on the adhesion of *Pseudomonas aeruginosa*. **(A)** Adhesion quantified by crystal violet staining and expressed as absorbance at OD_600_. **(B)** Percentage inhibition of bacterial adhesion relative to the untreated *P. aeruginosa* control. **(C)** Representative photograph of the crystal violet-stained 96-well microtiter plate following the adhesion assay. Data are expressed as mean ± standard deviation (SD) with a sample size of three (*n* = 3). Statistical significance was assessed using a one-way analysis of variance (ANOVA) followed by Tukey’s multiple-comparison test. Asterisks denote significant differences compared to untreated *P. aeruginosa*: *P* < 0.05 (*). Hash symbols indicate significant differences relative to the GLU 1% group: *P* < 0.01 (##), *P* < 0.001 (###), and *P* < 0.0001 (####). Dollar symbols represent significant differences compared to the antibiotic-treated group: *P* < 0.0001 ($$$$); ns indicates not significant.

### 3.8 Effect of *B. longum* and *L. plantarum* -derived Cell-free supernatants on *P. aeruginosa* twitching motility

*Bifidobacterium longum*-derived cell-free supernatants (CFS) significantly inhibited the twitching motility of *P. aeruginosa* (Figure 9A). Regardless of the fermentation substrate or post-processing treatment, all *B. longum*-derived preparations markedly reduced bacterial motility compared to the untreated control (one-way ANOVA, *P* < 0.0001), with inhibition values typically ranging from 60 to 85%. Arabic gum-derived CFS consistently demonstrated high antimotility activity. Increasing the Arabic gum concentration from 1 to 2% did not significantly enhance inhibition, and both neutralized CFS (CFS-N) and pH-matched controls (CFS-pH) maintained comparable activity, indicating that the inhibitory effect of *B. longum*-derived AG metabolites was largely preserved following pH modification. Greater variability was observed among baobab- and glucose-derived preparations. Although all treatments significantly inhibited motility, BB 2% CFS and its processed derivatives generally exhibited lower inhibition than the corresponding Arabic gum treatments. In contrast, the glucose pH-matched control (GLU 1% CFS-pH) produced the highest inhibition among the glucose-derived preparations and significantly exceeded several baobab-derived treatments. A direct comparison between AG 2% CFS and its neutralized counterpart, AG 2% CFS-N, revealed a slight numerical decrease in motility inhibition (81.0 ± 0.5% compared to 74.2 ± 0.4%). Nevertheless, this difference did not reach statistical significance after applying Tukey’s multiple-comparison adjustment. These results suggest that the neutralization process did not substantially affect the antimotility activity of AG 2%-derived CFS, indicating that the inhibitory effect was largely maintained post pH neutralization. Overall, these findings indicate that *B. longum*-derived metabolites strongly suppress *P. aeruginosa* twitching motility, and that this activity is largely preserved after neutralization or pH matching, particularly for Arabic gum-derived fermentations. On the other hand, the cell-free supernatants derived from *Lactiplantibacillus plantarum* significantly inhibited the twitching motility of *P. aeruginosa* compared to the untreated control, as demonstrated by a one-way ANOVA (*P* < 0.0001) (Figure 9B). However, unlike *B. longum*, the extent of inhibition was markedly affected by both substrate concentration and pH modification. The parent *L. plantarum* culture and most cell-free supernatants (CFS) generated with 1% Arabic gum, 1% baobab, or glucose exhibited high levels of motility inhibition (approximately 70–90%), which were comparable to each other, indicating effective suppression of bacterial motility by these treatments. Conversely, increasing the Arabic gum concentration to 2%, especially following neutralization (CFS-N) or pH matching (CFS-pH), progressively diminished the antimotility activity. Among all *L. plantarum*-derived treatments, AG 2% CFS-pH showed the weakest inhibition, suggesting that the inhibitory metabolites produced during fermentation with higher Arabic gum concentrations were more susceptible to pH modification. Unlike the significant changes observed with Arabic gum, the baobab-derived and glucose-derived CFS maintained relatively consistent inhibitory activity regardless of neutralization or pH matching, with no significant differences among most treatments. These findings indicate that the influence of pH modification on *L. plantarum*-derived metabolites is substrate-dependent and particularly pronounced during fermentation with higher concentrations of Arabic gum. Overall, these results demonstrate that *L. plantarum*-derived metabolites effectively inhibit *P. aeruginosa* twitching motility, although their activity is more sensitive to substrate concentration and pH modification than that of *B. longum*.

**FIGURE 9:**
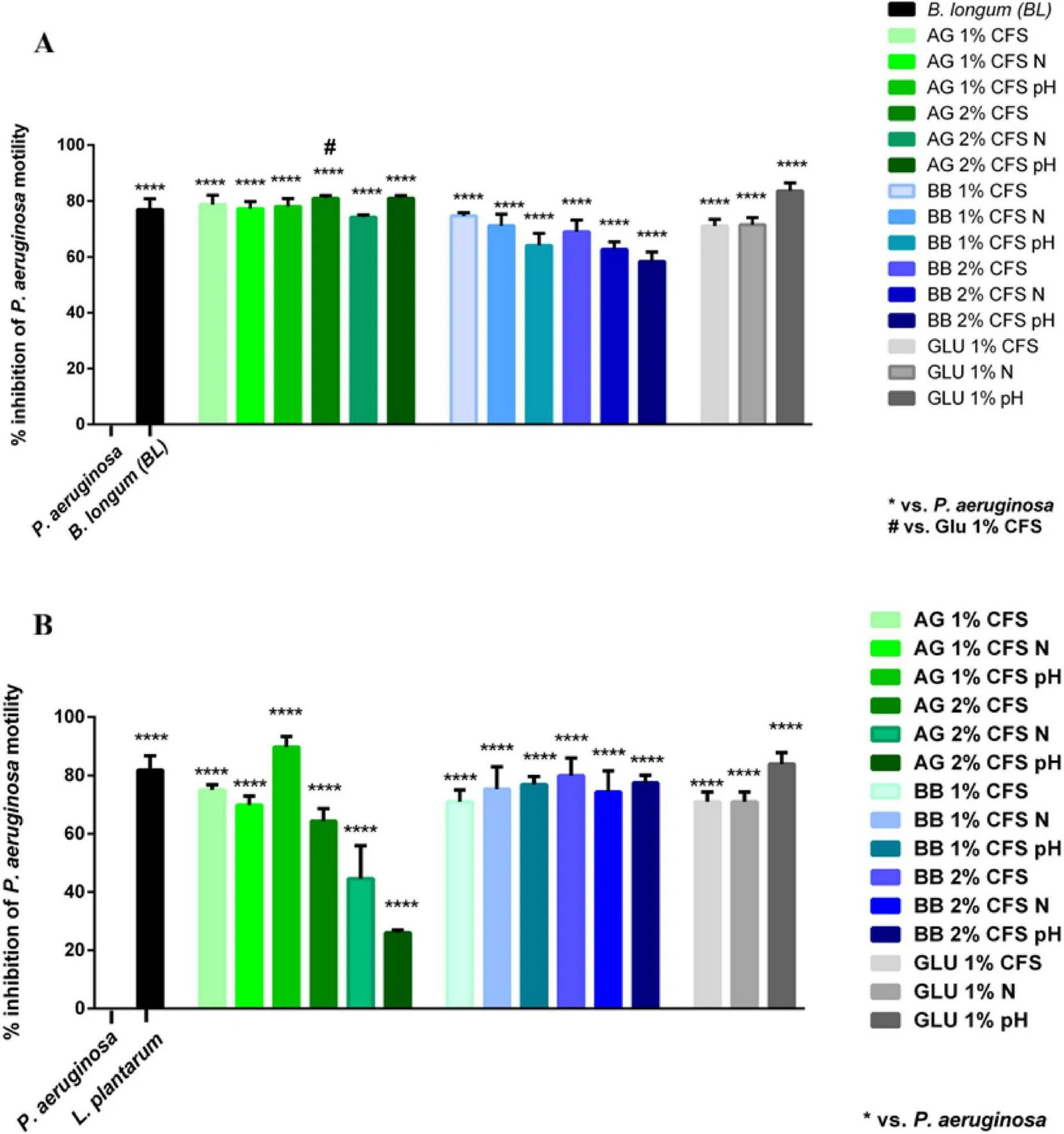
**(A)** Effect of *Bifidobacterium longum*-derived- cell-free supernatants (CFS) on the twitching motility of *Pseudomonas aeruginosa*. CFS were produced following fermentation with Arabic gum (AG), baobab (BB), or glucose (GLU). CFS-N represents neutralized cell-free supernatants (adjusted to pH 7.0 with NaOH), whereas CFS-pH represents pH-matched controls prepared by adjusting fresh sterile medium to the pH of the corresponding original CFS. Motility inhibition was expressed as the percentage reduction in twitching-zone, diameter relative to the untreated *P. aeruginosa* control. Data are presented as mean ± SD (*n* = 3). Data are presented as mean ± SD. Statistical significance was determined using one-way ANOVA followed by Tukey’s multiple-comparison test. Asterisks indicate significant differences relative to the untreated *P. aeruginosa* control: *P* < 0.0001 (****). Hash symbols indicate significant differences relative to the GLU 1% CFS group: *P* < 0.05 (#), and *P* < 0.001 (###), ns, not significant. **(B)** Effect of *Lactiplantibacillus plantarum*-derived cell-free supernatants (CFS) on the twitching motility of *Pseudomonas aeruginosa*. CFS were produced following fermentation with Arabic gum (AG), baobab (BB), or glucose (GLU). CFS-N represents neutralized cell-free supernatants (adjusted to pH 7.0 with NaOH), whereas CFS-pH represents pH-matched controls prepared by adjusting fresh sterile medium to the pH of the corresponding original CFS. Motility inhibition was expressed as the percentage reduction in twitching-zone diameter relative to the untreated *P. aeruginosa* control. Data are presented as mean ± SD. Statistical significance was determined using one-way ANOVA followed by Tukey’s multiple-comparison test. Asterisks indicate significant differences relative to the untreated *P. aeruginosa* control: *P* < 0.0001 (****). Hash symbols indicate significant differences relative to the GLU 1% CFS group: *P* < 0.05 (#); ns, not significant.

## 4 Discussion

The current study integrated comparative CAZyme profiling with experimental fermentation assays to examine the impact of structurally diverse prebiotic substrates on probiotic metabolism and the subsequent inhibition of *P. aeruginosa* virulence. Comparative genomic analysis revealed that *B. longum* and *L. plantarum* possess significantly larger repertoires of glycoside hydrolases (GHs) compared to *P. aeruginosa*, with *L. plantarum* exhibiting the highest number of glycosyltransferases (GTs). Conversely, *P. aeruginosa* encodes relatively few carbohydrate-active enzymes for the degradation of complex plant polysaccharides. These genomic distinctions imply that the probiotic strains are better equipped to utilize Arabic gum and baobab-derived carbohydrates than the opportunistic pathogen, aligning with the functional diversity documented in the CAZy database and comparative bacterial genome analyses (Lombard et al., 2014). These findings align with the established ecological specialization of gut commensals. Species within the genera *Bifidobacterium* and *Lactobacillus* have developed extensive systems of carbohydrate-active enzymes, facilitating the breakdown of dietary polysaccharides that are not digested in the upper gastrointestinal tract (Flint et al., 2012; Turroni et al., 2018). Glycoside hydrolases initiate the decomposition of complex glycans into fermentable oligosaccharides, while glycosyltransferases are involved in carbohydrate metabolism, cell-wall biosynthesis, and exopolysaccharide production. These functions collectively support adaptation to carbohydrate-rich intestinal environments (Drula et al., 2022; Lombard et al., 2014). Conversely, *P. aeruginosa* predominantly utilizes simple carbon sources and host-derived nutrients, possessing a more limited array of enzymes for plant polysaccharide degradation. This reflects its ecological role as an environmental opportunistic pathogen rather than a specialized gut carbohydrate fermenter (Drula et al., 2022). These genomic traits elucidate why prebiotic substrates are typically metabolized selectively by beneficial microorganisms rather than pathogenic bacteria (Gibson et al., 2017).

Our CAZyme findings align with prior comparative genomic studies, which have shown that Bifidobacterium species exhibit some of the highest densities of carbohydrate-active enzymes within the human gut microbiota, indicative of their adaptation to a variety of dietary glycans (Stover et al., 2000; Turroni et al., 2018). Similarly, *L. plantarum* is noted for its significant carbohydrate metabolic versatility, attributed to its expanded GH and GT families, which facilitate the utilization of numerous plant-derived carbohydrates (Martino et al., 2016; Siezen and van Hylckama Vlieg, 2011). In contrast, the relatively limited CAZyme profile of *P. aeruginosa* observed in this study is consistent with its role as an environmental opportunistic pathogen rather than a specialized polysaccharide fermenter (Stover et al., 2000). Notably, the CAZyme analysis accurately predicted the experimental fermentation outcomes observed in this study. Both probiotic strains effectively utilized Arabic gum and, to a lesser extent, baobab, whereas these substrates did not support pathogen growth to the same extent. Consequently, the computational analysis provided mechanistic support for the selective fermentation experiments and demonstrated that genomic carbohydrate-utilization potential translated into measurable biological activity. These findings further reinforce the current definition of prebiotics as substrates selectively utilized by beneficial microorganisms to confer health-related effects (Gibson et al., 2017).

The in vitro fermentation experiments revealed that the composition of the substrate significantly affected the growth and metabolic activity of probiotics. While glucose resulted in the highest increase in optical density for both probiotic species, Arabic gum, particularly at a concentration of 2%, elicited the most biologically significant response in *B. longum*. This was evidenced by an increase in viable counts and the greatest reduction in culture pH. These results suggest that turbidity alone may not be the most informative measure of prebiotic performance. True prebiotic efficacy is contingent upon the selective stimulation of metabolically active bacterial populations, rather than merely an increase in biomass, aligning with the current ISAPP definition of prebiotics (Gibson et al., 2017; Roberfroid, 2007). The observed discrepancy between OD_600_ and viable counts is biologically plausible. Optical density measures the total suspended biomass, encompassing viable cells, non-viable cells, extracellular polymers, and cell aggregates, whereas colony-forming units quantify only living bacteria capable of replication (Madigan et al., 2016; Sutton, 2011). The fermentation of complex polysaccharides often stimulates exopolysaccharide synthesis and bacterial aggregation, both of which can affect optical density independently of cell viability (Lebeer et al., 2008; Zeidan et al., 2017). Consequently, the modest increase in OD accompanied by higher viable counts observed with Arabic gum suggests that this substrate preferentially promoted bacterial viability and proliferation rather than merely increasing culture turbidity. The pronounced reduction in culture pH further supports the active fermentation of Arabic gum by *B. longum*. Members of the genus Bifidobacterium metabolize complex carbohydrates primarily through the fructose-6-phosphate phosphoketolase pathway (the bifid shunt), producing acetate and lactate as the principal fermentation end products (Pokusaeva et al., 2011; Turroni et al., 2018). These organic acids reduce environmental pH and contribute to colonization resistance by suppressing the growth and virulence of competing microorganisms (Flint et al., 2012; Rivière et al., 2016). Therefore, the greater acidification observed following Arabic gum fermentation suggests enhanced production of fermentation-derived metabolites that likely contributed to the downstream inhibition of *P. aeruginosa* observed in the present study. In contrast, glucose exerted a stronger influence on the growth of *L. plantarum* than on *B. longum*. This observation aligns with the remarkable metabolic versatility of *L. plantarum*, which efficiently metabolizes readily fermentable carbohydrates through glycolytic pathways and rapidly converts them into lactic acid (Martino et al., 2016; Siezen and van Hylckama Vlieg, 2011). Nevertheless, despite promoting greater bacterial growth, glucose did not consistently generate the strongest anti-*P. aeruginosa* activity, indicating that the biological activity of probiotic fermentation products depends more on metabolite composition than on bacterial biomass alone. These findings show that Arabic gum acts as a selective prebiotic by supporting active probiotic groups and boosting acid production during fermentation, especially in *B. longum.* This aligns with earlier research that highlighted the bifidogenic effects of Arabic gum in both lab fermentation systems and human studies (Ali et al., 2009; Calame et al., 2008). Additionally, the current results suggest that Arabic gum’s targeted probiotic stimulation produces fermentation products that can reduce several virulence-related traits of *P. aeruginosa*, forming the basis for the following CFS experiments.

One key finding of this study is that probiotic-derived cell-free supernatants (CFS) significantly inhibited the growth and virulence of *P. aeruginosa*. By using untreated CFS, neutralized CFS, and pH-matched controls, we could separate the effects of acidic fermentation products from those of pH-independent bioactive metabolites. This approach offers insights often missing in previous studies on probiotic antimicrobial activity (Bermudez-Brito et al., 2012; Oelschlaeger, 2010). The analysis of untreated CFS, neutralized CFS, and pH-matched controls revealed that the acidification associated with fermentation significantly influenced the suppression of *P. aeruginosa* growth. In various conditions, fresh medium adjusted to the same acidic pH as the corresponding CFS replicated or surpassed the inhibitory effect of the CFS, whereas neutralization markedly diminished antibacterial activity. These results suggest that low pH, likely resulting from organic acid production during fermentation, accounted for a considerable portion of the observed growth inhibition. However, certain neutralized preparations, particularly some *B. longum*-derived baobab CFS, retained residual inhibitory activity, indicating that non-acidic fermentation products may also play a role under specific conditions. As individual metabolites were not quantified or chemically characterized in this study, their contribution remains uncertain and necessitates targeted metabolomic investigation. Similar findings have been noted for several *Lactobacillus species*, where neutralization reduced but did not eliminate antimicrobial activity, indicating the presence of other bioactive compounds beyond organic acids (Hussein et al., 2025; Sharma and Im, 2018). Both *B. longum* and *L. plantarum* are known to produce various antimicrobial metabolites during carbohydrate fermentation, such as lactic acid, acetic acid, bacteriocins, hydrogen peroxide, biosurfactants, small antimicrobial peptides, and other low-molecular-weight compounds (Lebeer et al., 2008; Oelschlaeger, 2010). While organic acids lower pH and hinder bacterial growth, such probiotic-derived metabolites have been reported to affect bacterial membranes, metabolic pathways, quorum sensing, and biofilm-associated processes (Bermudez-Brito et al., 2012; Plaza-Diaz et al., 2019). The more pronounced response to Arabic gum compared to baobab under the current experimental conditions likely reflects differences in substrate accessibility for the selected probiotic monocultures, rather than an inherently lower prebiotic value of baobab. Arabic gum is abundant in branched arabinogalactan structures, which may be accessible to carbohydrate-active enzymes encoded by *B. longum* and *L. plantarum*. In contrast, baobab contains low-methoxylated pectic polysaccharides, whose efficient depolymerization may require specialized pectin-degrading microorganisms and community cross-feeding. Supporting this interpretation, Foltz et al. demonstrated that baobab fruit pulp stimulated acetate and propionate production in complex human fecal communities, with Bacteroidota identified as likely contributors to homogalacturonan degradation, and donor-dependent increases observed in bifidobacteria and lactobacilli (Foltz et al., 2021). Therefore, the relatively modest response to baobab in the present monoculture system does not contradict its reported prebiotic potential but suggests that its full fermentation capacity may be more effectively expressed within a diverse gut microbial community.

The differing responses extracted by glucose and Arabic gum are likely attributable to variations in carbohydrate metabolism. Glucose, as a readily fermentable monosaccharide, rapidly enhances probiotic growth. In contrast, the more complex structure of Arabic gum undergoes extended fermentation, resulting in a wider array of bioactive metabolites that can modulate bacterial virulence (Flint et al., 2012; Milani et al., 2017). Consequently, although glucose promoted greater probiotic biomass, Arabic gum generated fermentation products that consistently exhibited greater inhibitory activity across multiple *P. aeruginosa* virulence-associated assays. The findings suggest that the effectiveness of prebiotics depends on both the stimulation of probiotic growth and the quality of metabolites generated during fermentation. This highlights substrate-driven metabolism as a critical factor in anti-virulence activity.

In this study, rather than solely assessing bacterial growth, we investigated three critical virulence-associated phenotypes of *P. aeruginosa*: biofilm formation, surface adhesion, and motility. These phenotypes are integral to bacterial colonization, persistence, antibiotic tolerance, and chronic infection (Flemming et al., 2016; Moradali et al., 2017). Both Arabic gum- and baobab-derived probiotic cell-free supernatants (CFS) were found to reduce these virulence traits, with Arabic gum consistently exhibiting a more pronounced inhibitory effect. The consistent reduction observed across independent virulence assays indicates that probiotic fermentation products exert pleiotropic effects on *P. aeruginosa*, impairing bacterial attachment, surface colonization, and biofilm development simultaneously, rather than targeting a single pathogenic mechanism. Biofilm formation is a primary survival strategy of *P. aeruginosa*, providing protection against host immune defenses and reducing antibiotic penetration through the extracellular polymeric matrix (Flemming et al., 2016). In this study, the cell-free supernatant (CFS) derived from Arabic gum exhibited nearly complete inhibition of biofilm formation, while the CFS from baobab also reduced biofilm biomass, albeit to a lesser degree. These results indicate that probiotic fermentation products from both substrates disrupt the initial stages of biofilm establishment, rather than merely inhibiting growth. Similar anti-biofilm activities have been documented for probiotic cell-free supernatants, which impede *P. aeruginosa* biofilm formation by disrupting quorum sensing, extracellular polymeric substance production, and other virulence-associated pathways (Al-Saafin et al., 2023; Asadzadegan et al., 2023; Drumond et al., 2023). Collectively, these findings support the notion that metabolites derived from probiotics mitigate biofilm development through anti-virulence mechanisms in addition to their antimicrobial properties. A similar pattern was observed for bacterial adhesion, the initial step necessary for surface colonization and subsequent biofilm maturation (Islam et al., 2022). Arabic gum-derived CFS reduced *P. aeruginosa* adhesion to levels comparable to the antibiotic control, while baobab-derived CFS resulted in a moderate but measurable reduction. These findings suggest that probiotic fermentation products may interfere with bacterial surface interactions by altering cell-surface properties, reducing adhesin-mediated attachment, or disrupting extracellular matrix assembly (Lebeer et al., 2008; Plaza-Diaz et al., 2019). Motility was similarly reduced following treatment with probiotic CFS from both Arabic gum and baobab, with greater inhibition observed with Arabic gum. Since bacterial motility facilitates surface exploration, colonization, and early biofilm development, its inhibition further restricts infection establishment (Jahid et al., 2013). The observed simultaneous reduction in adhesion, biofilm formation, and twitching motility aligns with interference in the interconnected stages of *P. aeruginosa* pathogenesis. However, the molecular pathways responsible for these phenotypic effects were not directly investigated in this study. Specifically, the study did not quantify quorum-sensing signals or the expression of genes associated with the Las, Rhl, and Pqs systems. Consequently, while modulation of quorum-sensing and related virulence-regulatory pathways is a plausible mechanism, it remains a hypothesis rather than a confirmed molecular mechanism. Future research should assess the expression of key quorum-sensing genes, such as lasI/lasR, rhlI/rhlR, and pqsA/pqsR, in conjunction with genes associated with biofilm formation, adhesion, and type IV pilus, including pilA and pilT. Additionally, targeted metabolomic analysis of fermentation supernatants and quantification of quorum-sensing molecules would aid in identifying the bioactive metabolites and molecular pathways responsible for the observed anti-virulence phenotypes. Collectively, these findings support an anti-virulence mode of action for probiotic-derived fermentation products. Although Arabic gum consistently produced the strongest inhibitory responses, baobab also demonstrated measurable anti-virulence activity, indicating that both substrates possess functional prebiotic potential but differ in the spectrum and potency of the metabolites generated during probiotic fermentation. Such anti-virulence approaches are increasingly recognized as promising complementary strategies to conventional antibiotics, as they reduce bacterial pathogenicity while potentially imposing less selective pressure for antimicrobial resistance. Future research should expand upon these phenotype-level findings by incorporating integrated molecular and metabolomic analyses. Employing targeted qRT-PCR or transcriptomic profiling may elucidate whether fermentation-derived metabolites influence the Las, Rhl, or Pqs quorum-sensing networks and the downstream genes implicated in biofilm formation, type IV pili-mediated twitching motility, and surface attachment. Concurrent targeted and untargeted metabolomic analyses of cell-free supernatants would aid in identifying the organic acids and other bioactive metabolites linked to the observed inhibitory effects. Validation across multiple multidrug-resistant clinical isolates of *P. aeruginosa* is crucial for assessing the generalizability of the response. Additionally, investigations utilizing mixed microbial communities, gut-simulation systems, and suitable *in vivo* models are necessary to ascertain whether the substrate-dependent effects observed in the current monoculture system persist in more physiologically complex environments.

## 5 Conclusion

This study illustrates that the integration of genome-informed carbohydrate utilization prediction with experimental fermentation constitutes an effective framework for identifying microbiome-targeted strategies to mitigate bacterial virulence. Comparative analysis of CAZymes predicted that *B. longum* and *L. plantarum* possess significantly greater capacities to metabolize plant-derived polysaccharides compared to *Pseudomonas aeruginosa*, a prediction subsequently validated through fermentation experiments. Among the substrates assessed, Arabic gum consistently demonstrated the highest prebiotic functionality under the experimental conditions. Fermentation of Arabic gum preferentially enhanced probiotic metabolic activity, particularly in *B. longum*, facilitated greater acidification, and produced cell-free supernatants capable of suppressing multiple virulence-associated phenotypes of *P. aeruginosa*, including bacterial growth, biofilm formation, surface adhesion, and twitching motility. Although baobab also exhibited measurable prebiotic and anti-virulence activity, its effects were generally less pronounced in this monoculture fermentation model, suggesting that its full prebiotic potential may rely on the cooperative metabolism of more complex microbial communities. Comparison of untreated, neutralized, and pH-matched preparations revealed that fermentation-associated acidification significantly influenced the suppression of *P. aeruginosa* growth. The residual activity observed in certain neutralized CFS preparations implies that additional non-acidic fermentation products may also play a role under specific conditions. However, further characterization is necessary to identify these products and determine their relative contributions. These findings suggest that the biological activity of probiotic fermentation products is determined by substrate-driven metabolic outputs rather than bacterial biomass alone. Overall, this work establishes a functional link between genome-predicted glycan utilization, probiotic fermentation, metabolite production, and the attenuation of *P. aeruginosa* virulence. The integrated strategy presented here offers a valuable platform for developing microbiome-based anti-virulence approaches and supports the use of plant-derived prebiotic substrates, particularly Arabic gum, as promising adjuncts for controlling opportunistic pathogens without relying exclusively on conventional antibiotics.

## Data Availability

The raw data supporting the conclusions of this article will be made available by the authors, without undue reservation.
